# High-flow oxygenation therapy for a sedated elderly frail patient with hiccups undergoing transcatheter aortic valve implantation

**DOI:** 10.1186/s40981-025-00784-x

**Published:** 2025-04-21

**Authors:** Ryosuke Osawa, Takero Arai, Takashi Asai

**Affiliations:** Department of Anesthesiology, Dokkyo Medical University Saitama Medical Center, 2 - 1- 50 Minami-Koshigaya, Koshigaya, Saitama 343 - 8555 Japan

**Keywords:** High-flow nasal oxygenation therapy, Transcatheter aortic valve implantation, Conscious sedation

## Abstract

**Background:**

Transcatheter aortic valve implantation (TAVI) can be performed under sedation, but body movements may lower the efficacy of the procedure and may increase the risk of complications, such as cardiac tamponade. Additional sedatives and analgesics may be required to prevent body movements; this would increase the risk of upper airway obstruction and of respiratory depression. We report a frail patient with hypoxemia and hiccups, in whom high-flow nasal oxygenation facilitated TAVI by effectively inhibiting body movements and respiratory complications.

**Case presentation:**

In an 82-year-old patient with severe aortic stenosis, heart failure, hypoxemia, and hiccups, TAVI was planned under sedation with dexmedetomidine, fentanyl, and ketamine. High-flow nasal oxygenation effectively prevented hiccups and associated body movements, and prevented upper airway obstruction and respiratory depression, during TAVI.

**Conclusions:**

High-flow nasal oxygenation therapy is potentially useful during cardiac catheterization procedure under monitored anesthesia care, in elderly frail patients with reduced cardiopulmonary function.

## Background

Transcatheter aortic valve implantation (TAVI) has increasingly been used in patients with severe aortic valve stenosis and is the first choice in patients who would not tolerate surgical replacement, because of severe coexisting disease [1–6]. Although TAVI can be performed either under sedation or under general anesthesia, sedation may be more advantageous than general anesthesia in terms of a lower incidence of malignant dysrhythmia, a reduced cardiovascular depression, and a lesser requirement for inotropes and vasopressors [1, 2].

One major problem with sedation for cardiac catheterization procedure is that body movements due to pain, discomfort, sudden large inspiration (snoring), or hiccups may lower the efficacy of the procedure and may increase the risk of complications, such as cardiac tamponade and air embolism [4]. To reduce the body movements, additional sedatives and analgesics would be required, but this addition would increase the risk of upper airway obstruction and of respiratory depression, frequently requiring conversion to general anesthesia [1, 7].

High-flow nasal oxygenation, which delivers heated and humidified oxygen at flow rates as high as 70 l/min [8, 9], has been shown to be effective in oxygenation in patients undergoing gastrointestinal endoscopy under deep sedation [10]. In contrast, there has been only one study which assessed the efficacy of high-flow nasal oxygenation in sedated patients undergoing cardiac catheterization procedure [11]. The study [11], which compared the efficacy of conventional nasal oxygen (2 l/min) and high-flow nasal oxygenation (50 l/min; FiO_2_: 0.3), indicated that there was no significant difference in the primary outcome measure (arterial partial oxygen pressure), although it mildly reduced a secondary outcome measure (the incidence of hypoxemia (35% vs 24%)).

We report a case of a sedated frail patient with hypoxemia and hiccups, in whom high-flow nasal oxygenation facilitated TAVI by inhibiting body movements and avoided conversion to general anesthesia.

## Case presentation

A written consent was obtained from the patient for reporting this case.

An 82-year-old man (170 cm, 72 kg) was admitted to our hospital for treatment of severe aortic stenosis. The patient was extremely frail and hypoxemic, having hypertension, diabetes mellitus, and severe stenosis of the carotid artery, with a history of cerebral infarction, left paraplegia, and coronary artery bypass graft. Respiratory rates ranged from 16 to 18 breaths/min. No blood gas analysis was available.

Cardiac echography indicated severe aortic stenosis (the peak velocity: 5 m/s; aortic valve area: 1.0 cm^2^), with no apparent pulmonary hypertension (tricuspid regurgitation pressure gradient (TRPG): 21 mmHg). Laboratory examinations indicated acute cardiac failure (troponin I: 653.4 pg/ml (reference range < 20 pg/ml); brain natriuretic peptide (BNP): 540.5 pg/ml (reference range < 20 pg/ml); c-reactive protein (CRP): 2.66 mg/dl (reference range < 0.14 mg/dl)), without acute myocardial infarction (creatine kinase-MB (CK-MB): < 4 U/l (reference range < 25 U/l)). Cardiac surgeons judged the patient was not a suitable candidate for surgical aortic valve replacement, and thus planned to perform TAVI a few days later. Nevertheless, because the patient’s conditions had deteriorated with frequent occurrence of angina pectoris, continuous hiccups, and the arterial hemoglobin oxygen saturation (SpO_2_) being 88–92% (with oxygen at 3 l/min via a nasal cannula), cardiac surgeons decided to perform emergency TAVI.

Considering his condition, we decided to avoid general anesthesia and planned to provide monitored anesthesia care, but one possible problem in this patient was irregular movement of the heart due to hiccups would disturb TAVI. We felt that high-flow nasal oxygenation therapy would prevent deterioration of hypoxemia, may prevent upper airway obstruction, and also might reduce the degree of hiccups by producing positive end expiratory pressure effect [9, 12], thereby stabilizing the movement of the diaphragm. We planned to convert sedation to general anesthesia, if surgeons request us to minimize body movements during the procedure, or if there is a difficulty in maintaining oxygenation.

On arrival at the operating room, routine monitors, such as a blood pressure cuff, an electrocardiogram, and a pulse oximeter, were applied. The SpO_2_ was approximately 90% on room air, and the patient was having hiccups.

A nasal cannula (F&P Optiflow Trace Nasal Interface with an integrated CO_2_ sampling tube, Fisher & Paykel Healthcare, Auckland, New Zealand) was applied to the patient’s nose, and oxygen was provided at 30 l/min using a high-flow generator (Optiflow THRIVE nasal high flow therapy system, Fisher & Paykel Healthcare, Auckland, New Zealand). The tip of the CO_2_ sampling tube of the nasal cannula was directed to the patient’s mouth, to monitor carbon dioxide waveforms on a capnograph. Within a few minutes, SpO2 increased to 100%, and hiccups stopped completely. Intravenous and arterial lines were then established.

Sedation was induced with dexmedetomidine at 4 µg/kg/h, fentanyl 50 µg, and hydroxyzine 25 mg, and was maintained with dexmedetomidine at 0.4 µg/kg/h. To counter fentanyl-induced respiratory depression, 1 mg of levallorphan tartrate was administered, as a routine practice in patients undergoing cardiac catheter procedures in our hospital. Arterial blood gas analysis indicated that PO_2_ 300 mmHg and PCO_2_ 34.5 mmHg.

A central venous catheter was inserted into the right internal jugular vein under ultrasound guidance, and a temporary pacemaker lead was placed into the right ventricle. Heparin was injected intravenously and various guidewires and catheters were inserted from the surgical field. At the time of insertion of a sheath, ketamine 10 mg was injected intravenously, to minimize pain. This led to upper airway obstruction. Increasing oxygen flow from 30 to 35 l/min immediately relieved airway obstruction.

Propofol 10 mg or ketamine 10 mg was also injected intravenously, based on clinical conditions (such as the blood pressure, respiratory rate, and requirement of analgesic effect), during passage of a wire across the aortic valve, and during balloon aortic valvuloplasty and valve implantation under rapid ventricular pacing, but neither upper airway obstruction nor hypoxemia occurred. Because there was a significant paravalvular leak, post-dilation under rapid ventricular pacing was performed, which stopped paravalvular leak. After confirming no pressure gradient between the left ventricle and the aorta, protamine was injected and the procedure was completed. Throughout the procedure, dobutamine 1 µg/kg/min and noradrenaline 0.01–0.02 µg/kg/min were being infused, and no severe hypotension occurred. No hypoxemia occurred, and the PaO2/FiO2 (P/F) ratio in arterial blood gas analysis was approximately 300.

After the procedure, arterial blood gas analysis indicated that PO_2_ 156 mmHg and PCO_2_ 36.0 mmHg. Infusion of sedatives was stopped and the patient became awake. A few minutes after the replacing high-flow nasal cannula by a conventional Hudson facemask, hiccups recurred. No remarkable events occurred after the procedure, and the patient was discharged from the hospital, 6 days after TAVI.

## Discussion

We have reported a case of an elderly frail patient with hypoxemia and hiccups, in whom high-flow nasal oxygenation facilitated TAVI, by increasing SpO_2_, by instantly inhibiting hiccups, and by enabling us to provide sufficient doses of sedatives and analgesics to inhibit body movements responding to stimuli of the procedure, so that the TAVI was successful without converting sedation to general anesthesia.

Elderly people frequently have obstructive sleep apnea, and its incidence is up to 75% [13]. In addition, they are at increased risk of upper airway obstruction and respiratory depression, when they are sedated [14]. High-flow nasal oxygenation, initially used to treat patients with hypoxemic respiratory failure [15], inhibits the number of apnea and hypopnea during sleep in patients with obstructive sleep apnea [16]. The main reason for prevention of upper airway obstruction is that high-flow nasal oxygenation widens the pharyngeal space and elevates the epiglottis against the glottis, by producing a continuous positive pressure to the upper airway and a positive end-expiratory pressure [9, 12].

Pulmonary hypertension is frequently associated with severe aortic stenosis [17], and hypercapnia and acidosis caused by sedation may exacerbate pulmonary hypertension, leading to right ventricular failure. High-flow oxygenation may reduce the risk of exacerbation of pulmonary hypertension, by reducing upper airway obstruction [9] and by reducing the degree of hypercapnia [18, 19]. In addition, even when the patient becomes apneic, high-flow nasal oxygenation is effective in maintaining a high arterial oxygen saturation for a considerable duration of time [20].

Hiccups are usually minor and short-lived, but may last for months or years. Chronic hiccups may be associated with various cardiovascular disease, such as severe aortic valve stenosis [21], cardiac tamponade [22], myocardial infarction [23], aortic aneurysm or dissection [24], and dislodgement of pacemaker lead [25]. The etiology and mechanism of hiccups are not clearly understood, and chronic hiccups are frequently difficult to treat [26].

There have been no reports as to if high-flow nasal oxygenation can inhibit hiccups. A case report has indicated that high-flow nasal oxygenation may inhibit chronic coughing [27]. In the report of a patient with grade IV chronic obstructive pulmonary disease with intractable cough over 1 year who had been treated with home oxygen therapy (at 2 l/min), the start of high-flow nasal oxygenation (35 l/min, FiO2: 28%) was associated with complete disappearance of coughing 5 min later [27], possibly because of a positive end expiratory pressure effect, together with humidification of air.

In our patient, shortly after administration of high-flow nasal oxygenation, hiccups were completely inhibited, so that it became unnecessary to convert sedation to general anesthesia. The exact mechanism for this is not clear, but the positive end expiratory pressure effect produced by high-flow nasal oxygenation on the diaphragm was the likely reason to stop hiccups.

In conclusion, we believe that high-flow nasal oxygenation is potentially useful during cardiac catheterization procedure under monitored anesthesia care, in frail patients with reduced cardiopulmonary function.

## Data Availability

Not applicable.
